# Redefining the role of exercise: beyond medicine and polypill

**DOI:** 10.17179/excli2025-9114

**Published:** 2026-01-09

**Authors:** Kayvan Khoramipour, Susana López-Ortiz, Alejandro Santos-Lozano

**Affiliations:** 1 i+HeALTH Strategic Research Group, Department of Health Sciences, Miguel de Cervantes European University (UEMC), 47012 Valladolid, Spain

## ⁯

While the term "exercise is medicine" and "exercise is polypill" have been frequently used in literature, neither of them could adequately represent the complexity and multifaceted nature of exercise in health and disease management. In this paper, we highlighted the unique and broad range exercise benefits and advocated for a deeper understanding of its role in health promotion and disease prevention. We also emphasized that health care professionals should integrate exercise into routine healthcare practices as a fundamental element of comprehensive patient care. We propose a new term to be used in the texts “exercise is life vitalizer”.

Hippocrates stated two thousand years ago that *'walking is man's best medicine'*. He thus emphasized the early realization that physical activity is beneficial to health. Since then and more recently, there is robust evidence that physical activity reduces all-cause mortality and increases life expectancy (Martinez-Gomez et al., 2024[12]). Indeed, over the last two decades, numerous studies have enriched our knowledge of how exercise can be used as primary prevention method as well as a first-line treatment for several non-communicable diseases, including cardiovascular diseases, metabolic diseases and some cancers (Pedersen, 2019[15]). However, the role of exercise goes beyond symptom control, as it can improve physical fitness, mental well-being and quality of life, highlighting it as a key component within the health care system (Thompson et al., 2020[19]). 

Today, the World Health Organization (WHO) emphasizes the importance of physical activity for people's health and well-being, regardless of their condition. There is strong evidence that supports the major benefits of physical exercise as a non-pharmacological therapeutic intervention, but its application in standard clinical settings is still limited (Thornton et al., 2016[20]). This issue is because it is difficult and time consuming (O'Regan et al., 2021[14]) for physicians to prescribe individualized exercise plans. In addition, little investment has been made worldwide in policies to promote physical activity and exercise programs (WHO, 2018[22]). On top of that, excessive dependence on pharmacological therapies has several barriers. Firstly, low medication compliance negatively affects health outcomes and may increase the onset of complications (Aremu et al., 2022[2]). Additionally, the medication-based approach of the healthcare system can shift the responsibility of health management from patients to physicians. Another important barrier is that those patients undertaking medication are usually less likely to adopt healthy lifestyle, as they believe mediations are substitute for a healthy lifestyle. These challenges underscore the urgent need to prioritize exercise as a core element of chronic disease prevention and management (Strain et al., 2024[18]).

To address this global health problem, the American College of Sports Medicine (ACSM) and the American Medical Association, founded the Exercise is Medicine (EIM) action in 2007. This initiative was developed to make physical activity assessment and exercise prescription standard practice in disease prevention and treatment within the clinical care (Santos et al., 2023[17]). Now, EIM aims to narrow the gap between medical care and community-based resources (ACSM, o.J.[1]). Since its origin, EIM has grown drastically and now exists in nearly 40 countries around the world. Its efforts include the development of evidence-based guidelines, training programs for healthcare providers and public awareness campaigns to highlight the critical role of physical activity in improving people's health (ACSM, o.J.[1]). 

### Exercise is more than a polypill 

A 'polypill' is usually known as a type of drug combination consisting of multiple active ingredients in fixed doses, known as a fixed-dose combination, to treat or prevent different non-communicable diseases that was first proposed by Wald and Law in 2003 (Wald and Law, 2003[21]). Researchers have applied this concept to exercise as it can be as effective as some polypills and provide a bord range of health benefits even without side effects associated with pills (Naci and Ioannidis, 2015[13]). For instance, exercise (odds ratio, OR: 0.89) affects odds of mortality similar to statins (OR: 0.82), beta blockers (OR: 0.85), angiotensin converting enzyme inhibitors (OR:0.83), and anti-platelets (OR:0.83) in coronary heart disease. Exercise also reduces mortality odds more effective than drug in stroke (Naci and Ioannidis, 2015[13]).

Unlike drugs, which tend to focus on specific pathways, exercise has multisystemic effects on cardiovascular, metabolic, musculoskeletal and brain health. Through the release of exerkines (defined as signaling molecules released in response to acute and/or chronic exercise) (Chow et al., 2022[6]), which play a key role in mediating the systemic effects of exercise (Chow et al., 2022[6]), affects not only the musculature but also more distant organs such as the brain, liver, heart or adipose tissue (Chow et al., 2022[6]), promoting anti-inflammatory, metabolic and regenerative responses ref. 

Regular exercise influences several hallmarks of aging (i.e. macromolecular damage, dysregulated stress responses, disruption of proteostasis, metabolic dysfunction, epigenetic drift, inflammation and stem cell depletion) helping to prevent age-related chronic diseases and maintain functional capacity (Goh et al., 2023[8]). In addition, as clearly confirmed by several studies, exercise could improve hallmarks of health such as resilience, hermetic regulation, repair and regeneration, local homeostasis, and regulate multiple circuits alone with many more health related advantages (Qiu et al., 2023[16]). 

One of the most outstanding benefits of exercise is its role in mobilizing stem cells, essential for tissue repair and regeneration. High-intensity exercise facilitates the release of stem cells -such as hematopoietic stem cells and endothelial progenitor cells from the bone marrow into the peripheral blood, significantly increasing their availability in the bloodstream (Baker et al, 2017[3]). This process is supported by the increased expression of angiogenic factors during exercise, which improves blood flow in injured or inflamed areas (Berlet et al., 2022[4]). In addition, aerobic exercise can increase the regenerative function of stem cells by boosting blood flow, increasing tissue permeability and stimulating their migration and adhesion to damaged tissue. Exercise plays a vital role in remodeling of the extracellular matrix, facilitating stem cell homing, adhesion and integration into tissues (Berlet et al., 2022[4]). Exercise activates signaling pathways such as the AKT pathway that promote stem cell proliferation and support tissue regeneration. In addition, exercise-induced metabolic changes, including increased oxygen consumption and a shift from anaerobic glycolysis to oxidative phosphorylation, improve stem cell function in their microenvironment (Chen et al., 2022[5]). The production of antioxidants during exercise further improves stem cell survival rates in damaged areas, while modulating immune responses and regulating microRNAs involved in cell proliferation and migration (Marino et al., 2021[11]).

The effects of training go beyond regeneration, as it significantly promotes the formation of new cardiomyocytes and stimulates the proliferation of existing cells. This process, accompanied by angiogenesis and apoptosis inhibition, which activate endothelial progenitor cells and promote capillary and cardiomyocyte formation in a manner that is proportional to exercise intensity (Marino et al., 2021[11]). In addition, voluntary exercise increases the proliferation of neural progenitor cells in the brain and supports nervous system regeneration by activating different neural stem cell types (Larrick and Mendelsohn, 2020[9]). Exercise could also rejuvenate stem cells and prevent biological aging. Exercise has been shown to restore the regenerative capacity of muscle stem cells (i.e. satellite cells) improving their functions ((Larrick and Mendelsohn, 2020[9]). In addition, there is evidence that exercise may trigger the conversion of normal cells into stem cell like cells, a possibility that should be investigated further (Liu et al., 2023[10]). Research has also shown that exercise can induce epigenetic changes with the ability to transfer to the next generation, what is known as transgenerational epigenetic inheritance (Cintado et al., 2024[7]).

This broad range of exercise effects highlights the relevance of moving away from the traditional terms 'exercise is medicine' or 'exercise is a polypill' towards a more comprehensive concept that recognises the full spectrum of its physiological, metabolic, and regenerative effects. We propose the term *'exercise is a life vitalizer'* to capture its comprehensive ability to enhance health, prevent disease, and promote longevity, not only at the individual level but also through its potential influence on future generations via transgenerational epigenetic inheritance. This approach can inspire a health system and a social culture of proactive health and resilience that benefit individuals, communities and future generations. Furthermore, to overcome current barriers to integrating exercise into routine healthcare, medical education systems should provide more detailed courses to train physicians in prescribing exercise programmes. Health insurance systems could also offer incentives to physicians who prescribe exercise instead of medication whenever possible.

## Declaration

### Competing interests

The author declares no competing interests.

### Artificial Intelligence (AI) - assisted technology

We did not use any AI during preparing this paper. 
